# Prevalence and predictors of sub-optimal medication adherence among patients with severe mental illnesses in a tertiary psychiatric facility in Maiduguri, North-eastern Nigeria

**DOI:** 10.11604/pamj.2015.21.39.6664

**Published:** 2015-05-21

**Authors:** Abdu Wakawa Ibrahim, Shuaibu Yahya, Sadique Kwajafa Pindar, Musa Abba Wakil, Adamu Garkuwa, Shehu Sale

**Affiliations:** 1 Department of Mental Health, College of Medical Sciences, University of Maiduguri, Borno State, Nigeria; 2 Department of Community Medicine, College of Medical Sciences, University of Maiduguri, Borno State, Nigeria; 3 Department of Clinical Services, Abubakar Tafawa Balewa University Teaching Hospital, Bauchi, Bauchi state, Nigeria; 4 Department of Psychiatry, Bayero University Kano, Kano State, Nigeria

**Keywords:** Mental illnesses, Sub-optimal adherence, Maiduguri, Nigeria

## Abstract

**Introduction:**

Sub-optimal adherence constitutes a significant impediment to the management of severe mental illnesses (SMIs) as it negatively impacts on the course of the illness and the treatment outcome. In this study, the levels of adherence, prevalence and the predictors of sub-optimal adherence were assessed in a sub-Saharan African setting.

**Methods:**

Three hundred and seventy (370) respondents with diagnoses of schizophrenia, bipolar disorder or severe depression were randomly enrolled and interviewed at the out-patient department of the Federal Neuropsychiatric Hospital, Maiduguri in northeastern Nigeria. An anonymous sociodemographic questionnaire and a clinical proforma designed by the authors, Oslo social support scale and the 8-item Morisky Medication Adherence Scale (MMAS-8) were used for data collection.

**Results:**

The prevalence of sub-optimal adherence was 55.7%. The independent predictors of sub-optimal adherence were; seeking for traditional/ spiritual treatment (Odds Ratio (O.R.) = 6.523, 95% C.I. = 3.773 - 11.279, P = < 0.001), male gender (O.R. = 3.307, 95% C.I. = 1.907 - 5.737, P = < 0.001), low levels of insight (O.R. = 1.753, 95 C.I. = 1.220 - 2.519, P = 0.002), and low social support levels (O.R. = 1.528, 95% C.I. = 1.097 - 2.129, P = 0.012).

**Conclusion:**

Based on the outcome of the study, we recommend the development of psycho-educational programmes on adherence and the active involvement of the relations and significant others in the management of patients with SMIs in sub-Saharan Africa.

## Introduction

The severe mental illnesses (SMIs) are characterized by a chronic, and sometimes, fluctuating course as well as their association with significant functional impairments/disabilities [1–4]. The main aims of managing such disorders are to achieve and maintain remission of symptoms and, to reduce the accompanying impairments [5]. Psychotropic medications together with other non-pharmacological modalities constitute the cornerstones in the management of these disorders as espoused by the biopsychosocial model [6]. Since the SMIs are chronically disabling, their management specifically with medications are also longstanding and in some cases lifelong. A key determinant of the success of pharmacotherapy in patients with SMIs is their adherence to the medication regimen, which is defined as the extent to which medication intake behaviour corresponds with the recommendations of the health care provider [7–10]. Hence, non-adherence to medications (treatment) is the degree to which the patient does not carry out the clinical recommendations of a treating physician. Non-adherence to medications is a complex and multidimensional healthcare problem as it constitutes a major obstacle to translating treatment efficacy in research settings into effectiveness in clinical practice [11–13]. Research evidences abound that have shown its clinical significance on the course of the illness and treatment outcomes [14–16]. In addition, there can be a profound impact on the cost of care, as well as significant impediments to the patients´ long term adaptations, including the social, vocational and academic functioning [17, 18].

Globally, non-adherence rates among patients with severe mental illness ranged between 30% and 65% [19–21]. In schizophrenia, the average rate is about 50%, with a range of 4% to 74% [22–24]. In bipolar disorders, the non-adherence rate to long term prophylactic pharmacotherapy was between 20% and 66% [25–27]. In unipolar depression, the estimated non-adherence rates ranged from 13% to 52.7% [28–31]. The reported rates for non-adherence were 41.2% in Ethiopia and 74% in Egypt on the African continent [32, 33]. In Nigeria, the recorded rates of non-adherence among patients with SMIs ranged from 48% to 55.5% in Southern Nigeria, and 49.4% in Kaduna and 34.2% in Jos, North-western and North-central Nigeria respectively [34–37]. Sub-optimal adherence in psychiatric populations may be attributed to multifactorial influences Such as; age, gender, poor insight, negative attitude towards medications, shorter duration of illness, poor therapeutic alliance and poor social support [19, 21, 27, 34]. Africans, have their psychological and social peculiarities with regards to mental illnesses and such variables could affect their adherence to pharmacotherapy in the context of severe mental illnesses. Each African setting has its own peculiarities in terms of belief systems and social support as well as their effects on health seeking and adherence. To the best of our knowledge, this is the first study from North-eastern Nigeria that addresses this topical issue among patients with SMIs as a prototype sub-Saharan setting. It sought to ascertain; 1) the levels of adherence to pharmacotherapy among patients with severe mental illnesses and 2) determine the sociodemographic and clinical predictors of sub-optimal adherence among the subjects.

## Methods

### Study design and setting

This was a hospital-based cross-sectional study conducted at the outpatient department of the Federal Neuropsychiatric Hospital, Maiduguri, Borno State, Nigeria. As a matter of policy, all diagnoses made in the institution were according to the tenth edition of the International Classification of Diseases and health-related disorders (ICD - 10) criteria. Clinically generated data for each subject enrolled were matched to the ICD -10 criteria by Consultant Psychiatrists for quality assurance purposes.

### Participants

The sample size was calculated using a prevalence rate of 49.4% for non-adherence among patients with mental illnesses in northern Nigeria as reported by Adeponle et al. (2009), which was set at 95% confidence interval and 0.05 degree of precision [36]. This yielded a minimum sample size of 380 but a total of 390 respondents were interviewed in order to enhance the power of the study. Hence, one hundred and thirty (130) subjects each, with the diagnoses of; schizophrenia, bipolar disorder and severe unipolar depression were enrolled for the study. Patients were randomly selected using the table of random numbers during their respective clinic visitations. The eligibility criteria were: A diagnosis of Schizophrenia, bipolar disorder or severe unipolar depression, had been on medications for at least 6 months, adults above the age of 18years, and who granted consent. The exclusion criteria were: current florid psychopathology capable of impairing response, and comorbid psychoactive substance use or physical disorders. For the purpose of screening out those with significant psychopathology, mental state examination was conducted on all the participants at the index contact. Based on the outcome of this clinical evaluation alone, those that could not be engaged are excluded. While the exclusion of those with a comorbid physical disorder was based on previous clinical documentation, symptoms at presentation, general physical and relevant systemic examinations. All of the above clinical processes were independently conducted by two of the investigators.

### Measures

Data collection spanned between February and August, 2014, and the following standardized quantitative assessment tools were used: **(i)** Socio demographic questionnaire designed by the authors that solicited for age, sex, marital status and occupational status by using the social class stratification by Borofka and Olatawura [38]. This system classified individuals based on their occupations into: social class I (highly skilled respondents like Doctors, Lawyers, etc), social class II (intermediate skilled respondents like technicians, nurses, etc), social class III (low-skilled respondents like drivers, junior clerks, etc), social class IV (unskilled respondents like petty traders, messengers, etc) and finally social class V (unskilled respondents). It also asked about the respondents´ beliefs about the aetiology of mental illness. **(ii)** Clinical proforma also designed by the authors that sought for clinical information like diagnosis and duration of treatment as well as other forms of treatment sought and the degree of insight based on current assessment. For the assessment of insight the following dichotomous questions were asked and a score of 1 is assigned for yes and a score of zero for no for any item answered: Do you accept that you have an illness? Do you think that you require treatment? And, do you think you require your medications to stay well? The total score range between 0 and 3. A total score of zero is regarded as “no insight”, total score of 1-2 is “partial insight”, and a total score of 3 is interpreted as “full insight”. **(iii)** Oslo social support scale which is a 3-item scale that assesses the level of an individual's social support. The scale asks about the ease of getting help from neighbours, the number of people the subjects can count on when there are serious problems, and the level of concern people show in what the subject is doing. The instrument has been validated for use in Nigeria [39]. A sum index is obtained by adding the raw scores of the three items. The range is 3-14. The scores are interpreted as follows; 3-8 (poor social support), 9-11 (moderate social support), and 12-14 (strong social support) [40]. **(iv)** Morisky medication adherence scale (MMAS-8) which is an 8-item instrument which was developed by Donald Morisky to assess medication adherence among patients with different clinical conditions. In interpreting the outcome, the scores are graded as follows: <6 is low adherence, 6 to <8 is medium adherence and high adherence is equal to 8 [41]. For the purpose of this study, sub-optimal adherence is defined as MMAS-8 score of less than 8 while score of 8 is considered adherent. Similar cut-off score was adopted for non-adherence among hypertensive outpatients with comorbid psychiatric conditions in Ghana [42].

### Ethical consideration

Ethical clearance was obtained from the institutional review board of Federal Neuropsychiatric Hospital, Maiduguri. Written informed consents were also obtained from all the participants. In order to ensure confidentiality, codes were used for data entry and analysis.

### Data analyses

The data were analyzed using statistical package for social sciences (SPSS) version 20. Descriptive statistics were used to represent the characteristics of the participants. Bivariate analyses were used to explore the associations between the psychosocial variables and sub-optimal medication adherence among the participants. Binary logistic regression was then conducted to determine the independent predictors of sub-optimal adherence among the subjects. Sub-optimal adherence was used as independent variable while the factors found to be significant on bivariate analysis were used as covariates. Significance was computed at p < 0.05, two-tailed.

## Results

Of the 390 respondents enrolled, the data of 370 respondents were finally analyzed yielding an overall response rate of 94.87%. The data of 20 respondents were not analyzed due to: refusal to grant informed consent (n=8), comorbid psychoactive substance use (n=5), presence of florid psychopathology, particularly, auditory hallucinations and delusions (n=5), and those whose questionnaires could not be analyzed due to missing data (n=2). In terms of the sociodemographic characteristics of the respondents: males constituted 56.5%, their ages ranged from 18 to 63 years with an average of 35.06 years (SD+ 9.63). Over 70% of the respondents were ≤ 40 years of age. Semi-skilled and unskilled workers as well as the unemployed constituted over 60% of the respondents and about 51% were unmarried. About 43% of the respondents believed that mental illnesses were caused by either demonic possession or retribution for ´sins´ committed by one´s ancestors. The clinical characteristics of the respondents revealed that about 63% 0f the respondents had their illnesses for less than or equal to 4 years and nearly 56% of them had sought for either traditional African/spiritual forms of treatment apart from the conventional (orthodox) care. About 31% had poor social support while 69% had moderate to strong social support. About 58% had full insight, while, about 18% and 24% had no and partial insights respectively.

### Levels of adherence and prevalence of sub-optimal adherence

One hundred and sixty eight (45.4%), thirty eight (10.3%), and one hundred and sixty four (44.3%) had low, medium and high levels of medication adherence respectively. The prevalence rate for sub-optimal adherence was 55.7% using an MMAS score of <8 as adapted for this study. The findings are depicted in Table 1.

**Table 1:** Levels of adherence of the participants (N = 370)

Levels of adherence	Frequency (%)
Low adherence	168 (45.4)
Medium adherence	38 (10.3)
High adherence	164 (44.3)

### Socio-demographic variables associated with sub-optimal adherence

Of all the sociodemographic variables analysed for association with sub-optimal medication adherence only gender (χ^2^=50.415, df=1, p=<0.001) and belonging to lower occupational classes (χ^2^=57.93, df=4, p=<0.001) were found to be statistically significant. These are presented in Table 2.

**Table 2:** Socio-demographic variables associated with sub-optimal adherence (N=370)

Variables	Non-adherent Frequency (%)	Adherent Frequency (%)	Total Frequency (%)	Statistics
**Gender**				
Male	150(72.8)	59(36.0)	209(56.5)	**χ**^**2**^**=50.417, df=1, p=<0.001**++
Female	56(27.2)	105(64.0)	161(43.5)	
**Age in years [Mean = 35.06 years ± 9.628 SD, Range = 18 - 63 years]**				
≤ 40 years	145(70.4)	126(76.8)	271(73.2)	χ^2^= 1.933, df=1, p=0.164
> 40 years	61(29.6)	38(23.2)	99(26.8)	
**Occupational class**				
Skilled	14(6.8)	58(35.4)	72(19.5)	**χ**^**2**^**=57.93. df=4, p=<0.001**++
Intermediate	29(14.1)	32(19.5)	61(16.5)	
Semiskilled	17(8.3)	12(7.3)	29(7.8)	
Unskilled	44(21.4)	17(10.4)	61(16.5)	
Unemployed	102(49.5)	45(27.4)	147(39.7)	
**Marital status**				
Married	103(50.0)	78(47.6)	181(48.9)	χ^2^=2.72, df=3, p=0.437
Single	64(31.1)	50(30.5)	114(30.8)	
Widowed	16(7.8)	9(5.5)	25(6.8)	
Divorced	23(11.1)	27(16.4)	50(13.5)	
**Aetiological beliefs**				
Psychological	108(52.4)	103(62.8)	211(57.0)	χ^2^=4.269, df=2, p=<0.118
Demonic	65(31.6)	38(23.2)	103(27.8)	
Retribution	33(16.0)	23(14.0)	56(19.2)	

++ **Statistically significant findings**

### Clinical variables associated with sub-optimal adherence

The clinical variables found to have statistically significant associations with sub-optimal medication adherence were: the diagnosis (χ^2^=44.217, df=2, p=<0.001), seeking for alternate forms of treatment (χ^2^=70.217, df=1, p=<0.001), low levels of social support (χ^2^=27.265, df=2, p=<0.001), and low levels of insights (χ^2^=24.705, df=2, p=<0.001). The findings are shown inTable 3.

**Table 3:** Clinical variables associated with sub-optimal adherence (N=370)

Variables	Non-adherent Frequency (%)	Adherent Frequency (%)	Total Frequency (%)	Statistics
**Diagnoses**				
Schizophrenia	99(48.1)	29(17.7)	128(34.6)	**χ**^**2**^**=37.279,df=2, p=<0.001**++
Bipolar disorder	54(26.2)	70(42.7)	124(33.5)	
Sev. depression	53(25.7)	65(39.6)	118(31.9)	
**Duration of illness (in years)**				
< 2 years	72(35.0)	52(31.7)	124(33.5)	χ^2^=0.677, df=2, p=0.713
3 - 4 years	58(28.2)	52(31.7)	110(29.7)	
> 5 years	76(36.9)	60(36.6)	136(36.8)	
**Forms of treatment sought**				
Tradnal/spiritual	155(75.2)	52(31.7)	207(55.9)	**χ**^**2**^**=70.217, df=1, p=<0.001**++
Orthodox only	51(24.8)	112(68.3)	163(44.1)	
**Level of social support**				
Poor	79(38.4)	34(20.7)	113(30.6)	**χ**^**2**^**=27.265, df=2, p=<0.001**++
Moderate	67(32.5)	39(23.8)	106(28.6)	
Strong	60(29.1)	91(55.5)	151(40.8)	
**Level of insight**				
No insight	49(23.8)	17(10.4)	66(17.8)	**χ**^**2**^**=24.705, df=2, p=<0.001**++
Partial insight	61(29.6)	29(17.6)	90(24.3)	
Full insight	96(46.6)	118(72.0)	214(57.8)	

++ **Statistically significant findings**

### Logistic regression analysis for variables associated with sub-optimal adherence

Logistic regression analyses revealed that only gender (Odds ratio (O.R.) = 3.307, p = <0.001, 95% C.I. = 1.907 - 5.737) and lower occupational classes (O.R. = 0.643, P = <0.001, 95% C.I. = 0.539 - 0.767) were the independent predictors of sub-optimal adherence. While the independent clinical predictors were; diagnosis (O.R. = 1.428, p = 0.037, 95% C.I. = 1.021 - 1.998), spiritual or traditional treatment seeking apart (O.R. = 6.523, p = <0.001, 95% C.I. = 3.773 - 11.279), poor social support (O.R. = 1.528, p = 0.012, 95% C.I. = 1.097 - 2.129), and low levels of insight (O.R. = 1.753, p = 0.002, 95% C.I. = 1.220 - 2.519). The findings are depicted in Table 4.

**Table 4:** Logistic regression analysis for variables associated with sub-optimal adherence

Variables	Standard Error	Exp (B) Odds ratio	95% C. I. Lower – Upper	P - value
Gender	0.281	3.307	1.907 - 5.737	<0.001++
Occupatn class	0.090	0.643	0.539 - 0.767	<0.001++
Diagnosis	0.171	1.428	1.021 - 1.998	0.037++
Treatment sought	0.279	6.523	3.773 - 11.279	<0.001++
Social support	0.169	1.528	1.097 - 2.129	0.012++
Level of insight	0.185	1.753	1.220 - 2.129	0.002++

++ **Statistically significant findings**

## Discussion

This study assessed the levels of medication adherence and the psychosocial predictors of sub-optimal adherence among patients with SMIs in a tertiary mental health facility in north-eastern Nigeria. Though, other studies by Adeponle et al. (2009) and Danladi et al. (2013), addressed this issue in northern Nigeria, theirs were among patients with discrete diagnosis, mainly schizophrenia [36, 37]. To the best of the authors’ knowledge, this is the first study that addressed this topical issue among patients with diagnoses that cut across discrete clinical groups in this part of sub-Saharan Africa. The adherence rates were 45.4% for low adherence, 10.3% for medium adherence, and 44.3% for high adherence. This translates to about every one out of two of the subjects enrolled for the study had low adherence. This rate for non-adherence falls within the range of 30% to 65% estimated in previous studies by Yang et al. (2012), and Kassis et al. (2014) [20, 21]. It is also similar to the rates of 48% and 49.4% reported by Adewuya et al. (2009) and Adeponle et al. (2009) in Nigeria [34, 36]. The prevalence rate of sub-optimal adherence was 55.7% as defined in the methodology of this study to be the cumulative total of low and medium adherence rates. The rate of high adherence of 44.3% recorded in this study was also within the range reported by Adewuya et al. (2009) [34].

Though, sub-optimal adherence rate is within the range of earlier quoted values, this figure is higher than the rates of 49.4% and 38% reported by Adeponle et al. (2009), and Danladi et al. (2013) in northern Nigeria where the participants had similar sociodemographic characteristics [36, 37]. It was, on the other hand, lower than the values of 66.9% and 74% reported in Egypt and India respectively [21, 30]. In explaining this discrepancy, we advanced two possible explanations; first methodological differences in the studies, such as the tools used for the assessment of adherence and the different cut-off values used for the definition of adherence and non-adherence. Secondly, most of the previous studies, used respondents with homogenous diagnosis, i.e., schizophrenia, bipolar disorder or unipolar depression discretely. In this study, the three heterogeneous groups were assessed which brought the peculiarities of the three conditions such as medication regimen(s) to bear simultaneously. Among the sociodemographic variables analyzed for possible association with sub-optimal adherence only gender and occupational class were independent predictors after logistic regression. Males were over 3 times more likely to be sub-optimally adherent when compared to females. Demoz et al. (2014) similarly found higher adherence rates among females in Jimma, Ethiopia [14]. In a comprehensive literature review, Lacro et al. (2002), concluded that gender was not a consistent predictor of non-adherence to treatment [22]. The higher adherence rate of 64% recorded in females could be attributed to the patriarchal nature of the study setting in which females are socially and culturally expected to be compliant, and this might positively affect their adherence in the therapeutic alliance.

Analysis of occupational class as an independent predictor of sub-optimal adherence showed that over 79% of the non-adherent subjects belonged to the semi-skilled, unskilled and unemployed classes. Over 55% of the adherent subjects on the other hand, were either skilled or intermediate skilled professionals. This finding is in tandem with that of Verdoux et al. (2000), who found lower occupational status to be a predictor of poor medication adherence among first admitted psychotic patients [43]. The plausible reasons for this are; (1) since affordability is critical to adherence with medications, those in the higher occupational classes might not be encumbered in this case, because of their economic strength. (2) They may also be better motivated to adhere to treatment protocols in order to retain their jobs. The independent clinical predictors of sub-optimal adherence in this study were; diagnoses, forms of treatment sought, levels of social support and insight. Based on the outcome, patients with a diagnosis of schizophrenia were over 3 times more likely to be sub-optimally adherent than those with either bipolar disorder or unipolar depression. This might be attributed to residual symptomatology and difficulties with interpersonal adjustment that may be commoner in schizophrenia than either bipolar disorder or unipolar depression as reported by Fenton et al. (1997) [44]. In terms of treatment sought, seeking for traditional African/spiritual treatments apart from the conventional/orthodox care increases the likelihood of sub-optimal adherence by over 6 times. This is because seeking for traditional or spiritual interventions are more often accompanied by mythical aetiological beliefs and doubts about the efficacy of the conventional modalities. Secondly, most of the traditional or spiritual healers in sub-Saharan Africa as a precondition for their intervention routinely ask their clients to discontinue any form of “western medication”. The additive effects of these factors negatively impacts on medication adherence.

In terms of social support as an independent predictor, those with low levels of social support were about 2 times more likely to be sub-optimally adherent. This finding is in tandem with that of Fenton et al. (1997), Bolkan et al (2013), Rabinovitch et al. (2013), and Razali et al. (2014) that have all shown significant relationship between poor social support and medication non-adherence among psychiatric patients [44–47]. The possible reasons in this scenario are: (a) the family serves as a cue to action or reinforcing factor for drug adherence for psychiatric patients, (b) based on the social drift hypothesis, the chronic and disabling nature of most SMIs have negative implications on the socioeconomic statuses of the patients, therefore, the onus of purchasing the medications lies solely on the family and other members of the patients’ social networks. Poor social support base, therefore, may be an obstacle to optimal adherence.

A critical look at the level of insight as an independent predictor showed that those with lower levels of insight were about 2 times more likely to have sub-optimal adherence. Insight is the degree of conscious awareness of the presence of an illness. Since adherence has a positive correlation with illness awareness and health seeking, it means insight is a determinant of the significance a patient attaches to any form of treatment. The lack of it, therefore, undermines adherence. This finding is in tandem with that of Yen et al. (2005), Lincoln et al. (2007), Rocca et al. (2008), and Mohamed et al. (2009), that all reported relationships between insight and medication adherence among psychiatric patients [48–51].

### Limitations of the study

The limitations of the study were; (1) this is a questionnaire based study; objective methods of assessing adherence such as pill counts and metabolite bioassay could have been more reliable indicators of sub-optimal adherence (2) the cross-sectional nature of this study cannot permit drawing causal inference.

## Conclusion

Over half of the participants in this study were sub-optimally adherent to their medications we, therefore, recommend: (1) developing psycho-educational programmes that will address the misconceptions about the aetiology and spectra of symptoms associated with severe mental illnesses, (2) the active involvement of relatives and significant others in treatment planning and management of the patients with SMIs.
